# Prevalence of non *Helicobacter pylori* gastric Helicobacters in Iranian dyspeptic patients

**DOI:** 10.1186/s12876-020-01331-x

**Published:** 2020-06-16

**Authors:** Shakiba Shafaie, Hami Kaboosi, Fatemeh Peyravii Ghadikolaii

**Affiliations:** 1 Department of Microbiology, Ayatollah Amoli Branch, Islamic Azad University, Amol, Iran; 2 Department of Biology, Qaemshahr Branch, Islamic Azad University, Qaemshahr, Iran

**Keywords:** Dyspepsia, Gastric, Non-*Helicobacter pylori* gastric *Helicobacter*

## Abstract

**Background:**

Non *Helicobacter pylori* gastric Helicobacters (NHPGHs) are associated with a range of upper gastrointestinal symptoms, histologic and endoscopic findings. For the first time in Iran, we performed a cross-sectional study in order to determine the prevalence of five species of NHPGHs in patients presenting with dyspepsia.

**Methods:**

The participants were divided into *H. pylori*-infected and NHPGH-infected groups, based on the rapid urease test, histological analysis of biopsies, and PCR assay of *ureA*, *ureB*, and *ureAB* genes. The study included 428 gastric biopsies form dyspeptic patients, who did not receive any treatment for *H. pylori*. The samples were collected and sent to the laboratory within two years. *H. pylori* was identified in 368 samples, which were excluded from the study. Finally, a total of 60 non*-H. pylori* samples were studied for NHPGH species.

**Results:**

The overall frequency of NHPGH species was 10 for *H. suis* (three duodenal ulcer, three gastritis, and four gastric ulcer samples), 10 for *H. felis* (one gastritis, three duodenal ulcer, and six gastric ulcer samples), 20 for *H. salomonis* (four duodenal ulcer, five gastritis, and 11 gastric ulcer samples), 13 for *H. heilmannii* (three gastritis, five duodenal ulcer, and five gastric ulcer samples), and 7 for *H. bizzozeronii* (zero gastric ulcer, two duodenal ulcer, and five gastritis samples).

**Conclusions:**

Given our evidence about the possibility of involvement of NHPGHs in patients suffering from gastritis and nonexistence of mixed *H. pylori* infection, bacteriological testing of subjects negative for *H. pylori* becomes clinically relevant and important. Our findings suggest *H. salomonis* has the highest rate among the NHPGH species in Iranian dyspeptic patients.

## Background

The number of discovered *Helicobacter* species has increased rapidly in the last decade. Up to now, more than 30 species have been characterized and well-recognized by microbiologists. *H. pylori* is the most recognized bacterium associated with dyspepsia in humans [1–4]. However, NHPGH species with typical spiral morphology has the ability to colonize the stomach of humans and animals. NHPGHs, by neutralizing the gastric acid, provide a suitable environment for their survival [5–7]. Studies have shown that NHPGHs are involved in gastritis among humans [8–10]. Gastritis may progress to gastric atrophy, intestinal metaplasia, and gastric cancer over time and result in precancerous lesions (similar to monoclonal lymphocytic proliferation), development of lymphoid follicles, and even primary gastric lymphoma, which develops only in some patients with gastritis. The incidence of these conditions varies relative to the multifactorial influence of host virulence and bacterial factors, which are dissimilar in different social and racial groups [11].

NHPGHs can affect the stomach environment through different mechanisms [1, 11, 12]. *H. suis* is capable of escaping the host’s immune system and shows a long-term presence in the stomach [13]. These bacteria contain genes, essential for their survival in the stomach environment. Also, NHPGH species, such as *H. bizzozeronii*, can be distinguished from *H. pylori* considering their higher metabolic flexibility in terms of energy sources and chain of electron transport [14].

*H. felis* species promote mucosal cytokines, which play a role in the development of gastric cancer [15]. Previous research has reported co-infections with two NHPGH species, i.e., *H. salomonis* and *H. heilmannii*, in dyspeptic patients [16, 17]. On the other hand, *H. heilmannii* may be more involved in the formation of gastric MALT lymphomas, compared to *H. pylori.* To our knowledge, *H. pylori* mainly covers the mucosal layer, whereas *H. heilmannii* invades deeply into the antral glands [18].

The lack of information about the topic of current research was frequently mentioned in national congresses and health ministry priority research list. There is no information about the prevalence of NHPGH species in Iran. In the present study, we report, for the first time, the frequency of NHPGH species in dyspeptic patients in Iran. In addition, the possible relationship between NHPGH species and histological findings was examined.

## Methods

### Patients and sampling

**Fig. 1:**
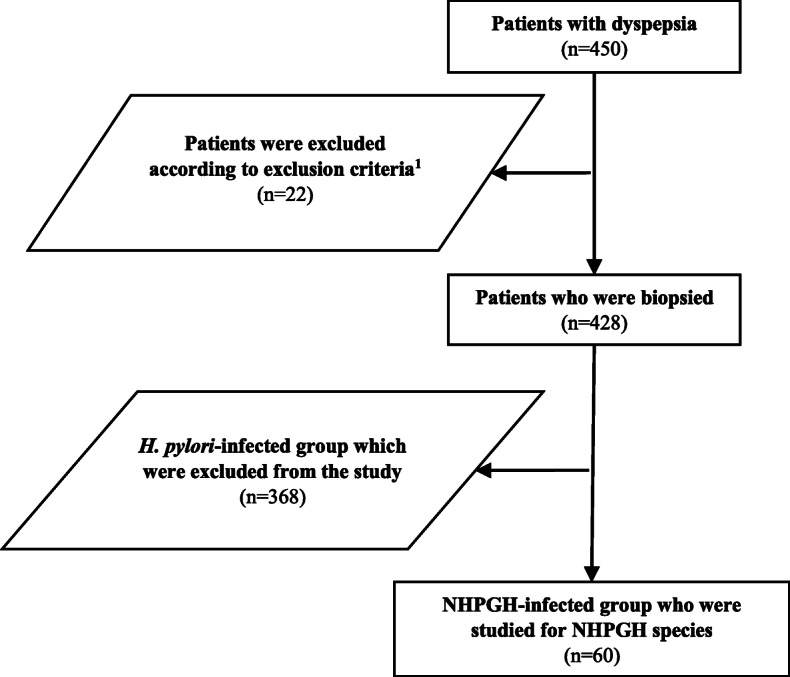
Flow chart of the study. ^1^The study’s exclusion criteria included I) receiving treatment for *H. pylori*, concurrent or recent antibiotic use such as metronidazole, clarithromycin, amoxicillin, tetracycline, doxycycline and other cephalosporin, II) histamine-2 receptor blocker or proton pump inhibitor therapy and bismuth compounds in the last four weeks; III) patients with regular use of NSAID; IV) patients with severe concomitant disease and V) patients with upper GI surgery

In this study, between March 2017 and February 2018, 428 dyspeptic patients, scheduled for upper gastrointestinal endoscopy (Mehrad Hospital, Tehran, Iran), were examined for *Helicobacter* infections using the Rapid Urease Test (RUT) and histological examination of stomach biopsies. According to the result of these tests, the samples were categorized into NHPGHs mono-infected (n, 60) and *H. pylori* mono-infected (n, 368) groups. The NHPGH mono-infected group comprised of 60 dyspeptic patients, who were studied for the prevalence of NHPGH species, while the *H. pylori* group included 368 dyspeptic patients, who were excluded from the study. A standard clinical pro forma was used to collect the demographic and clinical characteristics of NHPGH mono-infected patients via interviews. The study’s exclusion criteria included I) receiving treatment for *H. pylori*, concurrent or recent antibiotic use such as metronidazole, clarithromycin, amoxicillin, tetracycline, doxycycline and other cephalosporin, II) histamine-2 receptor blocker or proton pump inhibitor (PPI) therapy and bismuth compounds in the last four weeks; III) patients with regular use of NSAID; IV) patients with severe concomitant disease and V) patients with upper GI surgery. The participants signed the informed consent forms, and the Ethics Committee of Clinical Research approved the study protocol. The flow diagram of this study is shown in Fig. 1.

### Histological examination

The biopsy sections were embedded in 10% buffered formalin. Next, hematoxylin and eosin staining was applied to assess gastritis, while Giemsa staining was used to detect *Helicobacter* species. The histological patterns were classified as gastric ulcer, duodenal ulcer, and gastritis, using the updated Sydney system.

### DNA isolation

A Qiagen Genomic DNA Extraction Kit (BioFlux, USA) was used to isolate DNA from the stomach biopsies. Thereafter, DNA was resuspended in distilled water free of RNase/DNase (UltraPure).

### Molecular detection of NHPGH species

PCR assay was performed to detect NHPGH species, including *H. salomonis*, *H. bizzozeronii*, *H. heilmannii, H. felis*, and *H. suis* [19, 20]. Table 1 shows the primer sequences for NHPGH species. The amplification reactions were performed using 1X Reaction Buffer (0.2% gelatin, 16 mM of ammonium sulfate, 67 mM of tris/HCl, and 0.45% triton X-100), Taq DNA polymerase (one unit; Biotech International), deoxynucleotide triphosphates (200 mM each), 2 mM of MgCl_2_, oligonucleotide primers (10 pmol each), and 1 μL diluted DNA (typically a 1:10 dilution of the original sample at nearly 20–100 ng/μl); with a final volume of 50 μL. For every specific reaction, the amplification parameters are described below. A thermocycler (Perkin Elmer PE2400) was used to perform the reactions. In addition, Agarose mini-gel in TAE buffer (1 mM EDTA and 40 mM tris-acetate) was used to separate the PCR products. The products were then imaged under UV transillumination following ethidium bromide staining.

**Table 1:** PCR primers for NHPGH species genes amplification (in both Conversional and Real time PCR)

Species	Target Gene	Primer sequence	PCR Product Size (bp)	Reference
***H.suis***	*ureA*	FW (5′-CAC CAC CCC GGG GAA GTG ATC TTG-3′) RV (5′-CTA CAT CAA TCA AAT GCA CGG TTT TTT CTT CG-3′)	253	[20]
***H. bizzozeronii***	*ureA*	FW (5′-CGCTTT CAC CCC GGG GAA GTG ATC TTG-3′) RV (5′ TATCGCAACCGCAATTCACAACA-3′)	172	[19]
***H. felis***	*ureB*	FW (5′-TCCCACTACCGGGGATCGTG-3′) RV (5′ CAGCGGTTACAATCAAGCCCTCA-3′)	350	[19]
***H. salomonis***	*ureAB*	FW (5′-CTTTGGGTCTGTGCCTGCCTG-3′) RV (5′ CATCGCGGATAGTCTTACCGCCT-3′)	219	[19]
***H. heilmannii***	*ureA*	FW (5′-CTTTCTCCTGGTGAAGTGATTCTC′) RV (5′ CAGTTGATGGTGCCAAAG-3′)	368	[19]

For urease I reactions, the cycling conditions included three minutes of denaturation at 94 °C for 4 min; then 35 cycles at 94 °C for 10 s, at 52 °C for 20 s, and at 72 °C for 90 s; and a five-minute extension at 72 °C. In urease II reactions, the conditions were similar to those of urease I reactions with some modifications, i.e., 30 s of annealing and two minutes of extension at 42 °C and 72 °C, respectively. By analyzing the urease sequences from the strains and isolates, *ureA* gene regions, which were dissimilar in *H. felis*, *H. bizzozeronii*, and *H. salomonis* species*,* could be identified. Also, in Type-I PCR assay, the cycling conditions included five minutes of denaturation at 94 °C, followed by 35 cycles of amplification at 94 °C for 10 s, at 55 °C for 30 s, at 72 °C for one minute, and at 72 °C for four minutes.

### Real time PCR

Real-time PCR was performed using a Light Cycler 480 (Roche – Germany) detection system with the SYBR green I fluorophore. Reactions were performed in 20 μl (total volume) mixtures which included 5 μM SYBR green I PCR master mix 5 μl of each primer at a concentration of 5 μM, and 1 μl of the template DNA. Analyses were performed with a Light Cycler 480. The following protocol was used for 50 cycles consisting of 95 °C for 15 s, 55 °C for 15 s, and 72 °C for 30 s [21]. A melting curve analysis was performed following every run to ensure that there was a single amplified product for every reaction (Table 1). We used the Real time PCR alone as a conformity test for PCR (not a quantitative test). In other words, the main purpose of our experiment at the Real-Time PCR was to confirm our findings as we confirmed our positive ones. Thus, it was not aimed to distinguish the various species.

### Statistical analysis

Data were analyzed in SPSS v. 16.0 (SPSS Inc., Chicago, IL, USA). The Chi-square test was used to calculate the association between the presences of five non- *pylori Helicobacter* species in NHPGH-infected group. A *P*-value of < 0.05 was considered as statistically significant. Results are expressed as mean ± standard deviation for continuous variables (e.g., age) and number (percentage) for categorical data (e.g., gender).

## Results

### Demographic and clinical characteristics

**Fig. 2:**
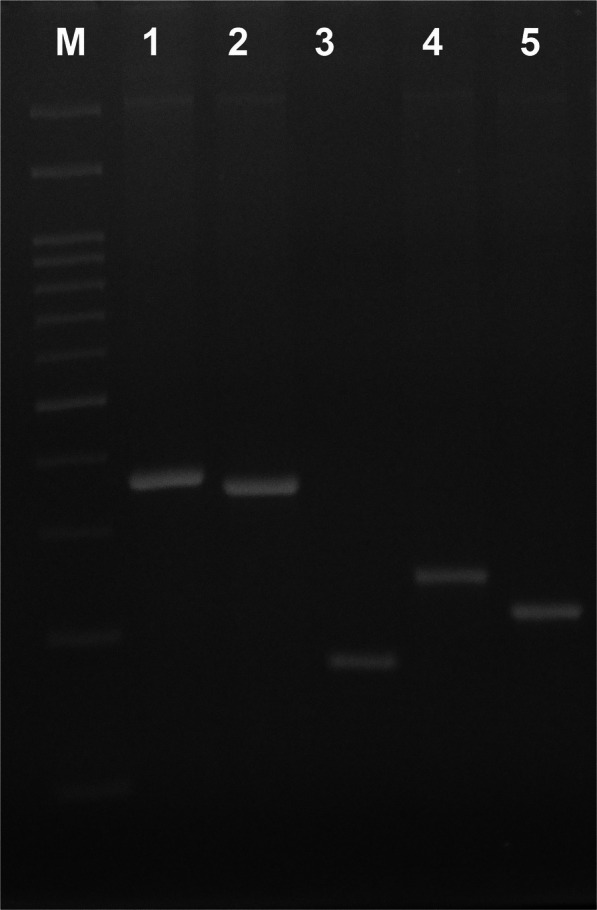
Agarose gel electrophoresis of the PCR products. Lane M is 100 bp (100 bp–3000 bp) DNA ladder (CinnaGen Co.). Lane 1 is DNA from representative NHPGH-infected patients for *ureA* positive *H. heilmannii* (368 bp). Lane 2 is DNA from representative NHPGH-infected patients for *ureB* positive *H. felis* (350 bp). Lane 3 is DNA from representative NHPGH-infected patients for *ureA* positive *H. bizzozeronii* (172 bp). Lane 4 is DNA from representative NHPGH-infected patients for *ureA* positive *H. suis* (253 bp). Lane 5 is DNA from representative NHPGH-infected patients for *ureAB* positive *H. salomonis* (219 bp)

Genomic DNA was collected from 60 NHPGH mono-infected patients. DNA was analyzed in all subjects. The NHPGH mono-infected patients’ demographic characteristics are presented in Table 2. Based on the findings, there was no significant difference (*p* > 0.05) among histological groups (i.e., duodenal ulcer, gastric ulcer, and gastritis) with respect to age and gender distribution. Among the 60 NHPGH mono-infected patients (which were negative for *H. pylori*) there was not co-infection with different species of NHPGH. In other words, was not observed more than one species of non-*H. pylori* in NHPGH mono-infected group. Agarose gel electrophoresis of the PCR products are shown in Fig. 2.

**Table 2:** Demographic data of NHPGH mono-infected patients enrolled

	Duodenal ulcer	Gastric ulcer	Gastritis
**Total: Female/Male** **(%)**	25: 10/15 (41%: 16%/25%)	15: 3/12 (25%: 5%/20%)	20: 9/11 (33%: 15%/18%)
**Age year (Mean ± SD**)	37.3	29.4	42.4

### Prevalence of non-*H. pylori* species in NHPGH mono-infected group

For comparative studies, NHPGH species were evaluated in histological groups, including duodenal ulcer (n, 25), gastric ulcer (n, 15), and gastritis (n, 20). The frequency of *H. suis* (n, 10), *H. bizzozeronii* (n, 7), *H. felis* (n, 10), *H. salomonis* (n, 20), and *H. heilmannii*-infected (n, 13) samples were compared with NHPGH-infected biopsies. Current findings showed that *H. salomonis* had the highest frequency rate, while the rate for *H. bizzozeronii* was the lowest. Based on the findings, none of the NHPGH species were associated with histological patterns (duodenal ulcer, gastric ulcer, and gastritis) (Table 3).

**Table 3:** Distribution of the five NHPGH species among the 60 pylori-negative patients

NHPGH species	Duodenal ulcer (*n* = 25)	Gastric ulcer (*n* = 15)	Gastritis (*n* = 20)	Total (*n* = 60)
***H. suis***	3	4	3	10
***H. bizzozeronii***	2	0	5	7
***H. felis***	3	6	1	10
***H. salomonis***	4	11	5	20
***H. heilmannii***	5	5	3	13
**Total**	17	26	17	60

## Discussion

Introduction of NHGPH species has provided researchers with an opportunity to determine the relationship between these species, which can colonize the animal and human guts, in order to better understand their effects on the host [22]. Within a short period of time after discovery of *H. pylori* by Marshall in 1983, scientists had understood that we have other members in this spiral type of bacteria causing the inflammation in human gastrointestinal route. The prevalence of NHPGH species in the gastric mucosa of humans and animals is diverse around the world [11, 23–26]. The main advantage of current research was to investigate in such naïve population with no information about prevalence and likely significant association between those strains and severe gastroduodenal diseases. Likely, in close future, current data can be a starting point for similar studies. In the present study, we applied the PCR assay to evaluate the frequency of NHPGH species in Iranian dyspeptic patients. In the literature, *H. suis* has been introduced as the most prevalent NHPGH species, colonizing the stomach of dyspeptic patients [13, 27]. According to the recent study by Nakagawa et al, *H. suis* was the main cause of chronic gastritis in individuals without *H. pylori* infection [28, 29]. According to previous studies, these species have a pathogenic potential due to the presence of gamma-glutamyl transpeptidase (*ggt*), their immune-suppressing properties, as well as outer membrane vesicles [13, 30]. Although pig farming, which is recognized as an important source of infection [31], is not permitted in Iran, there has been a relative increase in the frequency of *H. suis* (n, 10, 16%) among NHPGH species. Previous studies have shown that *H. suis* is a cause of acute inflammation in colonized patients in comparison with *H. pylori* and non-*pylori* Helicobacters, but such findings were not repeated in our examination [29, 32]. Similar to findings that De Cooman et al. released about pork meat consumption and the high risk of contaminated [20], we assume that this may happen in our population too, although technical and experimental errors may have been the source of our observation. Nevertheless, this rate of *H. suis* seems high among the Iranian individuals since pork is not in the regular dairy list. Indeed, the lack of knowledge about required duration time for transmitting the infection is still in place and we hope to have better insights in to this within the foreseeable future. On the other hand, *H. bizzozeronii* is the predominant NHPGH species in the canine stomach [11, 33]. There are multi potential factors involved in the virulence of *H. bizzozeronii*, including greater metabolic flexibility, genome plasticity, and harboring multiple methyl-accepting chemotaxis proteins [14, 16]. In addition, this species has been associated with severe dyspeptic symptoms [14]. In our study, we found seven dyspeptic patients infected with *H. bizzozeroni,* who claimed they were not in contact with dogs (as pets) since pet keeping is not common among Iranians. Therefore, *H. bizzozeroni* had the lowest prevalence among NHPGH species in our study population. However, further studies are needed to confirm this finding. *H. felis* infection is associated with reduced levels of interleukin-1β and tumor necrosis factor-α and increased level of interleukin-10, leading to the expression of key gastric mucosal cytokines and possibly gastric cancer [15]. The frequency of *H. felis* was 10 out of 60 (16%), thus we were unable to report any association between *H. felis* infection and histological report (*P* > 0.05). *H. salomonis* has been isolated from gastric biopsies of healthy dogs and humans. It has been also isolated from individuals infected with *H. heilmannii* [16, 17]. In the past, many studies reported that *H. heilmannii* infection is an example of zoonosis and we may have worrying report out of it in humans, but our results are quite contradictory [34]. In this study, *H. salomonis* was the most frequent NHPGH species (n, 20; 33%), while there is no similar study from the same location in Iran. In our study, the frequency of *H. heilmannii* was 13 out of 60 (21%). Since the transmission pathways for *H. salomonis* are unclear, we need to determine the probable source and route of transmission for this NHPGH species. On the other hand, *H. heilmannii* has been linked to gastritis, gastric ulcers and duodenal ulcers in humans [35]. We did not find significant association between presence of *H. heilmannii* infection and histological findings (*P* > 0.05). Indeed, this species can be the cause of apoptosis and angiogenesis in gastric MALT lymphoma [36]. Further studies with emphasis on molecular experiments are necessary to explain reports of such results.

### Limitations and future prospects

To the best of our knowledge, ours is the first cross-sectional study representing an Iranian population sample investigated for the clinical relevance of the five tested non-*pylori Helicobacter* species. Importantly, we found no mixed *Helicobacter* infections among NHPGH mono-infected group. We tried to make such big sample size in order to draw a good conclusion regardless significant finding or not. We think that our survey has good results, and it can be a reference study in future surveys in this country. However, our study had two limitations. The first limitation of our study was among *H. pylori* infected group (368 patients who were excluded from the study) that the possible co-infection with NHPGH different species was not investigated. This was due to the limited budget of our project. In this regard, we are starting the new project based on current panel of non-*pylori* helicobacters co-infection among *H.pylori* positive group. The second limitation was the need to establish a clear causative association between non-*pylori* helicobacters colonization and gastritis. Also, the significance of infection with NHPGH in terms of disease development could not be determined. The implications of this study are that non-*H. pylori* helicobacter species infection occurs in patients with abdominal pain or discomfort similar to *H. pylori* infection.

## Conclusion

Due to the difficulty associated with identification of non-*pylori* Helicobacters within routine laboratory tests, increased awareness of general health care and infectious diseases experts should be on the priority for decision-makers in hygiene and health in Iran. We conclude that infections with non-*pylori Helicobacter* species are candidates for further microbiological testing for targeted and improved clinical management. Nowadays the only fact we are confident of is that NHPGH species induce superficial inflammation in the gastric mucosa of colonized patients. The exact mechanism is not yet understood. However, further research is also necessary to clarify the epidemiology and pathogenesis of these mysterious bacteria.

## Data Availability

All original data and materials are available upon request from the corresponding author.

## Abbreviations

*H. pylori*: *Helicobacter pylori*
NHPGH: Non-*H. pylori* gastric Helicobacter
PCR: Polymerase chain reaction
*H. suis*: *Helicobacter suis*
*H. bizzozeronii*: *Helicobacter bizzozeronii*
*H. felis*: *Helicobacter felis*
*H. salomonis*: *Helicobacter salomonis*
*H. heilmannii*: *Helicobacter heilmannii*
NSAID: Nonsteroidal anti-inflammatory drugs
GI: Gastrointestinal
